# Advances in aqueous humor proteomics for biomarker discovery and disease mechanisms exploration: a spotlight on primary open angle glaucoma

**DOI:** 10.3389/fnmol.2024.1397461

**Published:** 2024-04-24

**Authors:** Vanessa M. Beutgen, Johannes Graumann

**Affiliations:** Institute of Translational Proteomics and Core Facility Translational Proteomics, Biochemical/Pharmacological Center, Philipps-Universität Marburg, Marburg, Germany

**Keywords:** aqueous humor (AH), proteomics, affinity proteomics, primary open angle glaucoma (POAG), biomarkers, LC-MS/MS, SOMAmer, proximity extension assay (PEA)

## Abstract

Altered protein levels in the aqueous humor (AH) may be a valuable source of novel biomarkers in neurodegenerative retinal disease. The proximity of this body fluid to the disease focus, and its corresponding enrichment for tissue specific proteins, renders it an excellent matrix to study underlying molecular mechanisms. Novel proteomic methods accordingly hold large potential for insight into pathologies based on the composition of the AH proteome, including primary open angle glaucoma (POAG). Recent mass spectrometry-based studies use novel approaches to tackle the challenges arising from the combination of low available sample volume and protein concentration, thereby increasing proteome coverage. But despite significant improvements in mass spectrometry (MS), a different class of proteomic technologies is poised to majorly impact the analysis of ocular biofluids. Affinity proteomic workflows, having become available commercially recently, have started to complement data obtained by MS and likely will grow into a crucial tool for ophthalmological biomarker research. This review highlights corresponding approaches in proteome analysis of aqueous humor and discusses recent findings on alterations of the AH proteome in POAG.

## Introduction

The aqueous humor (AH) is an ocular fluid supplying cells and tissues of the anterior chamber with nutrients. It is produced by the ciliary epithelium and drained via the trabecular meshwork (TM) and Schlemm’s channel. AH liquid biopsies are frequently performed during surgical intervention such as trabeculectomy or cataract surgery. The liquid may, however, also be safely obtained using hydro-dissection cannulas, enabling routine sampling (Kitazawa et al., 2017). AH contains a large number of proteins specific to ocular tissue and is thus exquisitely suited to screen for molecular changes in the eye. Despite being separated from blood by the blood-aqueous-barrier (BAB), proteins typically expressed in other organs may also be found in the AH (Coca-Prados, 2014; Yavrum et al., 2021; Wolf et al., 2023). This fact increasingly directs attention toward the AH proteome in the context of clinical biomarker research. Additionally, AH proteomics offers insight into the pathophysiology of neurodegenerative retinal disease. This review summarizes current advances in AH proteome investigation and their relevance to primary open angle glaucoma (POAG), as well as introduces relevant methodology.

## Deciphering the AH proteome

**FIGURE 1:**
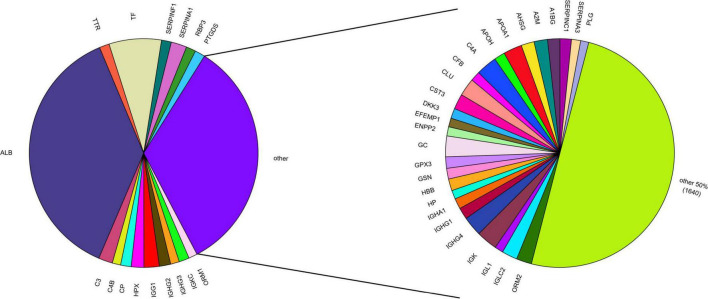
Protein abundance in the AH proteome based on mean PSM (peptide-to-spectrum matching) counts from AHP DB (Lee et al., 2024). The AH proteome is mostly composed of 17 high abundant proteins, representing 2/3 of the whole AH proteome **(left)**. Of these, albumin (ALB) alone accounts for ∼37%. Half of the remaining third is represented by 27 mid-abundant proteins **(right)**. The remaining 1,640 proteins from the AHP DB together account for only 1% of the total AH proteome, including many proteins differentially expressed in glaucoma (e.g., APOD, APOC3, GSTP1, NWD1).

Aqueous humor is collected in limited volumes of ∼50–150 μL per sampling with low protein concentrations of 0.1–0.6 μg/mL (Chowdhury et al., 2010; Bennett et al., 2011; Adav et al., 2018; Nikhalashree et al., 2019). This low protein yield renders proteomic analysis of AH challenging. Additionally, a high dynamic range of protein concentrations in AH (7 orders of magnitude), implies challenges similar to those faced in plasma proteomics (Yu et al., 2020). In AH, just 17 proteins constitute two thirds of the total protein content, with albumin alone accounting for approximately 37% (Figure 1). The remaining third again is dominated by 27 medium abundance proteins and low abundant proteins only represent 1% of the total (Yu et al., 2020). This protein concentration challenge is frequently tackled using depletion of high abundant proteins to enable detection of low abundance components (Lee et al., 2022). Functionally, AH proteins are mainly involved in immune response, inflammation, coagulation as well as energy metabolism (Yu et al., 2020), and it is considered strongly influenced by sex and ethnicity (Perumal et al., 2017; Haq et al., 2021; Vashishtha et al., 2023). General challenges in ocular liquid proteomics have recently been reviewed (Wolf et al., 2024).

Although AH may be safely sampled via paracentesis (Kitazawa et al., 2017), samples are usually collected during surgery. This commonly implies a lack of healthy controls, which is why AH samples from cataract patients frequently serve as controls. Moreover, treatment naive patients are often unavailable, further impeding unbiased analysis of AH proteomes. Treatments to reduce intra ocular pressure (IOP), commonly relying on timolol or xalatan, may also impact the AH proteome without appropriate control. These compounds were shown to in particular prevent complement activation and further protein level effects cannot be excluded (Blondin et al., 2003; Adav et al., 2018).

Current data bases cataloging the AH proteome contain 827 to 1,888 proteins identified using MS. Prominent resources are the EyeOme project under the auspices of the human proteome organization (HUPO) (Ahmad et al., 2018) and the recently released aqueous humor protein data base (AHP-DB), offering proteomic data deriving from 307 AH samples (Lee et al., 2024). A meta database combining data from multiple previous studies also exists (Yu et al., 2020). Beyond these catalogs, individual studies using label-free liquid chromatography-tandem MS (LC-MS/MS) in age-related macular degeneration (AMD) and Marfan Syndrome have reported proteome coverage in excess of 2,300 proteins (Coronado et al., 2021; Shi et al., 2024).

Although 2D-LC-MS/MS shotgun proteomics delivered good results in identifying 800+ protein groups in AH (Yu et al., 2020; Liu X. et al., 2021), data-independent acquisition (DIA) is increasingly applied in clinical proteomics and slowly replaces the more traditional data-dependent acquisition (DDA) approaches (Bader et al., 2023). By acquisition followed by informatic deconvolution of chimeric fragmentation data of all precursor ions within a defined mass-over-charge (m/z) window, DIA offers superior analysis depth and coverage. DDA in contrast only fragments and sequences defined precursor ions based on their intensity. Prefractionation of AH samples using high-performance liquid chromatography (HPLC) followed by MS/MS analysis in DIA mode have vastly increased the number of detectable proteins (Shi et al., 2024). Zhang and colleagues recently introduced a Streamlined Workflow based on Anchor-nanoparticles for Proteomics (SWAP) method in conjunction with DIA-MS, which identified ∼1,400 proteins from a minute 5 μL of sample (Zhang et al., 2023). The nanoparticles employed are functionalized with diverse surface coatings, allowing for differential protein enrichment from AH samples, and thus providing improved coverage of low abundant proteins. The approach is similar to the commercially available Seer Photograph platform (Seer, 2023).

Mass spectrometry (MS)-based AH proteomics, offering untargeted protein identification and high specificity, represents an outstanding asset. Achieving comprehensive protein coverage, however, commonly necessitates the depletion of high-abundant proteins and sample pre-fractionation. Consequently, and due to co-depletion, loss through surface coating in complex workflows and similar effects, detection of biomarkers in the low-abundant ranges is rendered challenging and inefficient.

The protein coverage of modern affinity-based assay platforms thus currently outperforms the analytical depth of state-of-the-art MS workflows in complex body fluids by a wide margin. Novel affinity proteomics discovery platforms, such as SomaScan 11k and Olink Explore HT, enable the analysis of thousands of protein targets in a semi-quantitative manner—without any sample pre-processing. While being largely developed for the analysis of blood-derived samples, the platforms offer access to sample matrices beyond those, including saliva (Scholtz et al., 2020), urine (Daza et al., 2023), ascites (Finkernagel et al., 2019) and many other bodily fluids including AH and vitreous humor (VH) (Lamy et al., 2020; Peng et al., 2023; Wolf et al., 2023).

As suggested by its moniker, the new SomaScan 11K platform, developed by Somalogic, provides ∼11,000 protein targets, encompassing half the human proteome, and provides the deepest coverage of all currently available methods (SomaLogic, 2024). For protein binders, SomaScan utilizes enhanced aptamers, short oligo single strand DNA (ssDNA) nucleotides, named slow off-rate modified aptamers (SOMAmers). Chemically modified to enhance affinity to protein targets, SOMAmers are conjugated to a fluorophore and a biotin tag via a photocleavable linker. Bound to streptavidin beads they capture proteins from the sample. Following biotinylation of captured proteins and cleavage of SOMAmer/protein complexes from the beads using UV light, the complexes are recaptured on fresh beads via the biotinylated proteins and SOMAmers eluted and analyzed on a microarray chip with signal intensity correlating to protein concentration. A first study using the SomaScan platform for ocular fluid analysis was conducted by Pessuti et al. (2023). AH from 28 patients with infectious uveitis and 29 samples from non-infectious uveitis patients was analyzed in comparison to 35 AH samples from cataract patients, using the SomaScan v3 Assay, identifying a minimum of 4,074 proteins across all groups.

An updated version of the SomaScan assay (v4.1) comprising 6,345 protein targets was also recently applied to analyze AH and VH liquid biopsies (Wolf et al., 2023). The authors were able to detect 5,953 proteins in the AH of healthy subjects, 5,887 of which replicated paired RNA-seq findings. This represents a significant improvement in analysis depth as compared to existing LC-MS/MS approaches. It is noteworthy, that the target menu of SomaScan v4.1 lacks coverage of various eye specific targets (Wolf et al., 2024), rendering likely an even higher number of protein detections from the sample type when subjected to the novel 11k panel.

A strong competitor in large panel affinity proteomics is the Swedish company Olink. Its Olink Explore HT platform covers approximately 5,400 protein targets and is based on proximity extension assay (PEA) technology (Olink, 2024). PEA uses two antibodies binding to distinct epitopes on a target protein. The antibodies covalently carry DNA probes that hybridize when in proximity. PCR amplifies the double-stranded sequences for readout using qPCR or Next-Gen Sequencing. Target protein-specific DNA barcodes comprised within the amplicon correlate with protein concentration, enabling relative protein abundance measurements. PEA offers minimal cross-reactivity, as well as high specificity and multiplexing capacity across a broad dynamic range, rendering it ideal for high-throughput analyses. While the latest Explore HT platform has yet to be applied to AH proteomics, the method has proven applicable using smaller panels and shown good detectability of AH proteins, as ∼70% of proteins form different Olink Target panels were detected in at least 30% of patients (Wilson et al., 2023). Olink affinity proteomic data from AH liquid biopsies was further demonstrated to be valuable for the prognosis of metastasis in uveal melanoma, an intraocular malignancy, rendering disease state accessible even in the absence of tumor biopsies (Wierenga et al., 2019; Peng et al., 2023). Here both the Olink Target Immuno-Oncology panel, targeting 90 marker proteins (Wierenga et al., 2019), as well as the more comprehensive Explore 1.5k panel, covering ∼1,500 protein targets (Peng et al., 2023) where employed to analyze AH samples. The studies identified new potential biomarkers for disease prognosis as well as stratification of metastasis, and highlight the efficacy of PEA-based methodology to comprehensively profile the AH proteome for biomarker discovery and mechanistic insight into the underlying disease.

## Correlation of serum and AH proteins: crossing the blood-aqueous-barrier?

Even though sampling of AH via paracentesis is considered safe (Kitazawa et al., 2017), protein biomarker analysis for routine diagnostics using blood-derived samples is even more so. Possible correlations between AH and blood proteins are thus of particular interest. The eye is an immune-privileged site, isolated from immune cells and other molecules from the blood-circulation by the blood-retina- and blood-aqueous-barrier (BRB, BAB) in protection from inflammation induced tissue damage. Nonetheless, passage of proteins through ocular barriers is possible and pathological conditions like elevated IOP may weaken them and facilitate reciprocal molecular transfer (Plange et al., 2012). Only limited overall correlation was, however, found between AH and serum proteins in patients suffering from various retinal disorders (Wu et al., 2020; Wilson et al., 2023). While complement component proteins C3 and C3a detected in AH did also not correlate well with serum levels, their ratio, was found to correlate exceptionally well between the two fluids (Hubens et al., 2021). Taken together, these observations, however, suggest insufficient suitability of AH biomarkers in blood-derived samples, limiting their suitability in routine diagnostics.

Another promising sample source for analyzing disease related proteome alterations and gaining insight into molecular disease mechanisms is the VH. Its proximity to the retina and optic nerve head renders it appealing for proteomic analysis. Unfortunately, obtaining samples of VH carries a significant risk for complications, resulting in limited availability for clinical studies. In contrast to limited exchange with blood via the BAB, however, studies have demonstrated the diffusion of vitreous proteins into the AH, alongside a significant correlation between the proteomic profiles of AH and VH (Wu et al., 2020; Wilson et al., 2023). In extension of that work it was only recently demonstrated that 87% of VH proteins were also detected in AH (Wolf et al., 2023). This provides an opportunity to examine differential protein expression linked to pathological changes in both the anterior and posterior portions of the eye through AH, thus potentially rendering accessible indicators of retinal neurodegeneration.

## Novel insight into glaucomatous changes of the aqueous humor proteome

Glaucoma is an umbrella term for a variety of conditions with a heterogenous presentation. They have in common a characteristic loss of retinal ganglion cells (RGCs) and associated optic nerve damage. Elevated intraocular pressure (IOP) is strongly correlated with, but not solely causative of glaucoma (Leske et al., 2001; Le et al., 2003). Untreated ocular hypertension (OHT) converts into glaucoma with a probability of 9.5% within five years of diagnosis (Kass et al., 2002). On the other hand, about one third of glaucomas are classified as normal tension glaucoma (NTG), never developing an increased IOP (Gutteridge, 2000). Occurring damage to the optic nerve head is hypothesized to be related to alterations in the translaminar pressure difference (TLPD) caused by alterations in CSF-mediated intracranial pressure (ICP) (Jonas et al., 2015). Abnormally low ICP and high IOP are thought to share similar pathogenic mechanisms affecting the lamina cribrosa. In fact, ICP is frequently reported to be lower in NTG compared to high tension glaucoma and healthy controls (Berdahl et al., 2008; Ren et al., 2010; Siaudvytyte et al., 2014). Accordingly, TLPD, referring to the pressure gradient between IOP and ICP (IOP minus ICP), is significantly higher in NTG patients (Ren et al., 2010; Siaudvytyte et al., 2014). Currently, IOP is the only risk factor accessible to treatment. Lowering IOP may stall disease progression and was found to reduce the five-year OHT conversion rate to 4.4% (Kass et al., 2002). Altogether, this implies a crucial role of AH dynamics in glaucoma pathogenesis and dysregulated AH fluctuation is frequently observed in high-pressure glaucoma. The etiology of increased intraocular pressure may, however, vary across different forms of glaucoma.

In primary open-angle glaucoma (POAG), AH efflux is impaired by enhanced extracellular matrix (ECM) deposition and altered actin cytoskeleton dynamics, causing a stiffening of TM tissue. In contrast, AH drainage in pseudoexfoliation glaucoma (PEXG) is obstructed by an accumulation of pseudoexfoliation material. Although different root causes may increase AH outflow resistance, they share the outcome of dysregulated AH dynamics. Frequent reports of AH proteome alteration associated with glaucoma underscore the significant role of AH dynamics in this disease and the analysis of its proteome consequently attractive to exploration of molecular mechanisms and identification of candidate drug targets.

A higher total protein concentration of AH in POAG has been reported repeatedly (Nikhalashree et al., 2019; Cappelli et al., 2020). AH proteome composition has also been found affected and might be reflective of pathological alterations in the TM, but also changes in the posterior of the eye (Kaeslin et al., 2016; Kodeboyina et al., 2021; Wolf et al., 2023). Given the diffusion of proteins from the posterior, including retina and optic nerve head, to the anterior chamber via the vitreous, protein profiles representative of pathological changes in the retina may also be observable. Differentially expressed proteins were found to correlate with visual field assessing parameters used in glaucoma diagnostics (Kodeboyina et al., 2021). For a number of proteins involved in neurodegeneration, immune response and metabolism this holds true for mean deviation (MD), pattern standard deviation (PSD), visual field index (VFI) and glaucoma Hemifield test (GHT) (Kodeboyina et al., 2021).

Currently, MS-based workflows represent the vanguard of AH proteomics. An overview of recent POAG studies on the AH proteome is given in Table 1. The highest protein coverage in a single experiment comparing proteomes of POAG and control samples, was accomplished to date by Adav et al. (2018). Using state-of-the-art LC-MS/MS, they identified 865 proteins including 150 differentially expressed ones. Among these, proteins associated with the complement system, neural degeneration, regulation of cholesterol esterification and apoptosis were significantly enriched (Adav et al., 2018). The reported alteration in complement component levels (down-regulation of C1q, C1r, C1s, C3, C4A, C4B, C5, C6, and C8) and lipid metabolism (upregulation of apolipoprotein A-IV), in particular, are frequently replicated observations. Another study, for example, identified 32 complement associated proteins in AH using LC-MS/MS, with C3, C4B and C4A as the most prevalent ones (Vashishtha et al., 2023). Of these, complement protein F2 was higher expressed in POAG samples, while C8G, C6, and complement factor H (CFH) were detected in lower concentration as compared to cataract controls. In contrast, C1q, C8B, C9, and C3 have been reported at higher levels in POAG (Kaeslin et al., 2016; Liu et al., 2020). Complement activation in AH of POAG patients was further investigated by analysis of C3a/C3 ratios (Hubens et al., 2021). An elevated ratio was observed in patients with progressing disease, while complement activation in stable POAG did not differ from cataract controls. The complement associated proteins C1s, C4A, C4B, as well as C8B were also observed to relate to abnormal PSD, VFI or GHT (Kodeboyina et al., 2021). Activation of the complement system via the three common pathways yields cleaved C3 and C5, leading to formation of the membrane attack complex (MAC). The complement system in POAG is broadly studied and its involvement in the pathogenesis has been extensively reviewed (Hoppe and Gregory-Ksander, 2024). Briefly, deposition of MACs and complement components in the glaucomatous retina have been repeatedly reported, also in relation to elevated IOP (Kuehn et al., 2006; Tezel et al., 2010; Jha et al., 2011), reaffirming a pivotal role in glaucoma pathogenesis and progression. In recapitulation, however, the aforementioned AH studies do not show a uniform expression profile of complement associated proteins. This may be attributable to IOP-reducing medication like timolol or xalatan, the enrolled patients were receiving (Blondin et al., 2003; Adav et al., 2018), potentially contributing to proteomic alterations beyond disease effects.

**TABLE 1:** Overview of recent AH proteome studies in POAG.

Goal of investigation	Samples (*n*)	Method details		Protein IDs	References
		Machine	Method		
Complement proteins in POAG	258 (196 CAT, 62 POAG)	Orbitrap Fusion Tribrid mass spectrometer (Thermo Fisher Scientific, Waltham, MA, USA)	LC-MS/MS, DDA	32	Vashishtha et al., 2023
Pathological processes and biomarker candidates	10 (5 POAG vs. 5 CTRL)	QExactive MS (Thermo Scientific) Easy-nLC nano-LC (Thermo Scientific)	LC-MS/MS, DDA, HRM-MS (SWATH)	448	Kaeslin et al., 2016
Proteome changes in POAG and PACG	9 (3 POAG, 3 PACG, 3 CAT)	NA	LC-MS/MS, MS^e^ mode	184 CAT 190 POAG 299 PACG	Nikhalashree et al., 2019
Exosomes in AH: sub-proteome	26 (16 POAG, 10 CTRL)	QExactive MS (Thermo Scientific) Easy-nLC nano-LC (Thermo Scientific)	iTRAQ (8-plex)	15	Mueller et al., 2023
POAG related proteomic changes	35 (12 CAT, 23 POAG)	Linear trap quadrupole Orbitrap MS	LC-MS/MS	175	Liu et al., 2020
Explore pathogenesis, Identify drug targets	20 (10 POAG, 10 CAT)	QExactive MS (Thermo Scientific)	Nano-HPLC-MS, LFQ	610	Liu A. et al., 2021
Correlation of AH proteome with visual field indices	49 (POAG only)	Orbitrap Fusion Tribrid (Thermo Scientific) Ultimate 3000 nano-UPLC (Thermo Scientific)	DDA LC-MS/MS	222	Kodeboyina et al., 2021
AH proteome glaucoma with and without PEX	29 (13 POAG (6/w PEX, 7/wo PEX) vs. 16 CAT (5/w PEX, 11/wo PEX))	Orbitrap QExactive (Thermo Scientific)	LFQ, DDA	269	Kliuchnikova et al., 2016
POAG pathogenesis and progression, treatment effects	10 (5 POAG, 5 CAT)	LFQ: QExactive (Thermo Fisher, Waltham, MA, USA) Dionex UltiMate 3000 UHPLC; MRM: TSQ Vantage triple quadrupole + EASY-nLC nano-LC (Thermo Scientific)	LC-MS/MS, LFQ, +MRM-MS	865	Adav et al., 2018
Proteomic alterations in POAG	47 (32 CAT, 15 POAG)	Orbitrap Fusion Tribrid mass spectrometer (Thermo Scientific) Ultimate 3000 nano-UPLC (Thermo Scientific)	LC-MS/MS	401	Sharma et al., 2018

HRM, hyper reaction monitoring; CAT, cataract; POAG, primary open angle glaucoma; PACG, primary angle closure glaucoma; CTRL, control; PEX, pseudoexfoliation; DDA, data-dependent acquisition; DIA, data-independent acquisition; SWATH-MS, sequential window acquisition of all theoretical mass spectra; iTRAQ, isobaric tags for relative and absolute quantitation.

Another protein class frequently linked to eye disease by proteomics is that of apolipoproteins. Apolipoprotein D (APOD), in particular, is found upregulated in AH of POAG patients (Kaeslin et al., 2016; Liu et al., 2020; Kodeboyina et al., 2021). It correlates positively with PSD, and negatively with VFI and MD. APOD further strongly associates with GHT. Its relation to these visual field indices hints at a role in glaucomatous neurodegeneration (Kodeboyina et al., 2021). APOD is a member of the lipocalin protein family with the main function of binding and transporting lipids and other small molecules (Rassart et al., 2020). It has been found upregulated in other neurodegenerative disorders, such as Alzheimer’s disease, Parkinson’s disease or multiple sclerosis, and is in general linked to aging and associated neurodegeneration (Dassati et al., 2014). APOD is suggested to be involved in neuroprotection based on its anti-oxidative and anti-inflammatory properties (Dassati et al., 2014). Other proteins belonging to this family, particularly APOA4, APOE, APOC1 and APOC3, are upregulated in AH of POAG patients as well (Kaeslin et al., 2016; Adav et al., 2018; Sharma et al., 2018), and have further been reported as upregulated in glaucoma in retina and/or VH (Mirzaei et al., 2017). Their link to neurodegenerative events in the retina renders them biomarker candidates for AH-based diagnostics, yet in turn potentially unfitting to a distinction between glaucoma and other neurodegenerative disease.

Beyond the frequently observed alteration in apolipoproteins and the complement cascade, various other glaucoma pathogenesis associated proteins were identified in AH. An accumulation of amyloid-beta (Aβ), associated with Alzheimer’s disease (Abyadeh et al., 2024), was for example observed (Adav et al., 2018), but the finding was not reproduced in another study (Cappelli et al., 2020). Artificially elevated Aβ in cerebrospinal fluid (CSF) was, however, demonstrated to directly transfer to AH in a transgenic AD mouse model, implying a potential for AD diagnostics (Kwak et al., 2020).

Furthermore, with GSTP1, a protein involved in the glutathione metabolism pathway associated with increased reactive oxygen species production and oxidative stress was identified as reduced in AH of POAG patients with cataract, (Liu A. et al., 2021). Low GSTP1 levels were discovered by MS-based proteomic analysis and subsequently verified by enzyme-linked immunosorbent assay (ELISA), validating this potential biomarker candidate and new treatment target. The authors identified a further 48 up- and 49 downregulated proteins in POAG that were mainly involved in the processes of inflammation, oxidative stress and ECM remodeling. These changes in oxidative stress response and inflammation are hypothesized to be linked to pathogenic alterations in the AH microenvironment. Other proteins associated with glycosylation, immune response, molecular transport and lipid metabolism (especially cholesterol homeostasis), such as NPC2, COL18A1, SERPINF2, NWD1 and KIAA0100, were found to be correlated with POAG odds ratios (Sharma et al., 2018). Receiver operating characteristic (ROC) analysis of these proteins further revealed promising potential for use as diagnostic biomarkers (AUC = 0.751 – AUC = 0.793).

Not only the liquid phase of AH is subject to disease related alterations, but cargo of AH derived extracellular vesicles (EVs) also differ between POAG and controls (Mueller et al., 2023). EV proteins from POAG patients and controls were labeled using isobaric tags for relative and absolute quantitation (iTRAQ) (Ross et al., 2004; DeSouza et al., 2005) and analyzed using high resolution MS. STT3B was found consistently downregulated in POAG EVs, a finding confirmed by western blot and ELISA. STT3B catalyzes lipid and protein N-glycosylation in the endoplasmic reticulum (ER), and is involved in the detection of misfolded proteins. Lower levels of STT3B may therefore indicate dysregulation of the unfolded protein response (UPR) as a coping mechanism for ER stress in TM cells, which is thought to be involved in glaucoma pathogenesis (McLaughlin et al., 2022).

## Conclusion

State-of-the-art MS proteomic workflows are increasingly expanding the knowledge about the AH proteome in health and disease. Several identified differentially expressed proteins bear the potential to serve as disease biomarkers in AH-based diagnostics. A transition from AH biomarkers to detection in blood-based samples, however, does not appear realizable as correlation between the two matrices proves insufficient. While sampling of AH is considered safe, it remains uncomfortable and is thus unlikely to be suitable for standard diagnostic testing. However, AH proteome studies shine in the discovery of disease mechanisms, with application in the development of causative treatments or neuroprotection through identification of candidate drug targets. Large potential for furthering such mechanistic insight from AH-analysis in glaucoma research lies with novel affinity proteomic technologies, which largely remain underutilized. The astounding capacity of such platforms to provide extensive protein coverage under conditions of extreme dynamic protein concentration range and using minimal amounts of precious sample material at high throughput is expected to significantly advance the field in the years to come.

## Author contributions

VB: Conceptualization, Visualization, Writing – original draft, Writing – review & editing. JG: Conceptualization, Funding acquisition, Supervision, Writing – review & editing.
